# Study on the Mechanism of Eerdun Wurile’s Effects on Post-operative Cognitive Dysfunction by the TLR4/NF-κB Pathway

**DOI:** 10.1007/s12035-023-03537-y

**Published:** 2023-08-07

**Authors:** Yun Qiao, Huiru Li, Yan Li, Enboer Su, Zhe Wang, Limuge Che, Yiri Du

**Affiliations:** 1 Department of Anesthesiology, The Affiliated Hospital of Inner Mongolia Medical University, Huimin District, Hohhot, 010059 Inner Mongolia Autonomous Region China; 2 Medical Innovation Center for Nationalities, Inner Mongolia Medical University, Hohhot, 010110 China; 3 Jinshan Economic Development Zone, Tumote Left Banner, Inner Mongolia Autonomous Region Jinshan Campus of Inner Mongolia Medical University, Hohhot City, China

**Keywords:** Post-operative cognitive dysfunction (POCD), Eerdun Wurile (EW) in Mongolian medicine, TLR4/NF-κB pathway, Lipopolysaccharide (LPS)

## Abstract

The object of our work was to observe whether the Mongolian medicine Eerdun Wurile (EW) improve postoperative cognitive dysfunction (POCD) by affecting the TLR4/NF-κB. Mice (6–8-week-old male C57BL/6 J) were selected to establish an animal model of POCD by combining intracerebroventricular injection of lipopolysaccharide and nephrectomy; EW formulation and EW basic formulation were administered intra-gastrically for 7 consecutive days. The cognitive performance was assessed by Morris water maze test. H&E staining was examined to detect alterations in hippocampal tissue. Immunohistochemical staining was performed to evaluate MyD88, NF-κB, TLR4, iNOS, and IBA-1 expressions; Western blotting and RT-qPCR were performed to evaluate MyD88, NF-κB, and TLR4. The expressions of IL-6, IL-1β, and TNF-α were evaluated by ELISA. Intracerebroventricular injection of lipopolysaccharide combined with nephrectomy induced cognitive dysfunction in mice, stimulated TLR4/NF-κB and microglia, and promoted the secretion of murine TNF-α, IL-1β, and IL-6. EW formulation and EW basic formulation treatment are able to suppress the TLR4/NF-κB pathway activation and microglia, and the serum cytokine secretions related to proinflammation, and restore the cognitive performance. EW formulation and EW basic formulation can improve POCD in mice, and TLR4/NF-κB pathway seems to be one of the important mechanisms in EW’s improvement of POCD.

## Introduction

Post-operative cognitive dysfunction (POCD) is a universal neurologic problem after operation and anesthesia. The main symptoms of POCD are cognitive dysfunction, inattention, abstract thinking, and memory impairment, often accompanied by mood swings and personality changes that seriously affect the patient’s quality of life [1]. POCD is more common in the elderly, and its occurrence is influenced by many factors, including age, gender, education level, type and number of anesthetics, and type of surgery [2]. Current data suggest that the prevalence of POCD within weeks following operation ranges from 10 to 54% [3]. Hence, it is imperative to explore novel drugs to treat and prevent POCD as soon as possible. This is why there is a critical need to search for new types of drugs to treat and prevent POCD.

Although the specific pathogenesis of POCD is unclear, numerous investigations have confirmed that inflammation plays a critical role in the progression and development of POCD [4, 5]. As a classical inflammatory pathway, TLR4/NF-κB has received growing interest in the study of POCD [6, 7]. TLR4, a toll-like receptor, is a type I transmembrane type protein widely distributed in brain neurons, microglia and astrocytes [8]. Lipopolysaccharide (LPS) is a specific exogenous ligand for TLR4, which can bind to the receptor TLR4 and activate the MyD88-dependent pathway as a downstream signaling to transduce inflammatory signals and also activate NF-κB (nuclear factor kappa B) [9, 10]. NF-κB is a critical nuclear transcription factor in early immune responses and cellular inflammation and promotes the secretion of cytokines for secretion, such as TNF-α (tumor necrosis factor-α), IL-6, IL-1β, and NO[11, 12]. Some investigations have also shown that surgical trauma causes TLR4 to bind to its corresponding ligand, resulting in the stimulation of inflammatory cytokines in the hippocampus via microglial activation [13], which may be closely related to POCD caused by surgical trauma.

Eerdun Wurile (EW) consists of 29 herbal medicines and is commonly applied for the treatment of nervous system dysfunction, including memory impairment, blurred facial expression, dizziness, and drowsiness [14]. Currently, the administration of EW in the therapeutic application of neuronal dysfunction is attracting increasing attention and many related studies have been conducted in China. It has been confirmed that EW improves neurobehaviors in rodents with middle artery occlusion-reperfusion injury and has specific preventive and therapeutic effects in treating stroke [15–17]. EW can also markedly decrease the cytokine expressions for inflammation, such as TNF-α, IL-6, and IL-1β in the brain, decrease apoptosis, and improve nervous system symptoms related to inflammation [15, 18, 19]. The EW basic formulation consists of 10 drugs for the treatment of nervous system diseases, selected from EW formulation based on Mongolian medical theory. Therefore, in theory, this basic formulation also has the effects of treating nervous system diseases and of reducing nerve inflammation.

Our previous studies have shown that EW improves post-operative cognitive dysfunction in rats through the signaling molecules PI3K and IRS-PI3K-ACT-GLUT4 signaling pathway downstream of insulin [20]. Traditional Mongolian medicine is illustrated by its multi-target and multi-component effects. In this study, to test whether EW can improve the development of POCD through the TLR4/NF-κB and to clarify whether EW basic formulation have POCD-improving effects, we observed specific mechanisms of action on POCD mice through EW and EW basic formulation. We intended to provide a rationale, novel therapeutic objectives, and new methods for the clinical application and prevention of POCD. At the same time, an in-depth study of the mechanism of function of EWs will reveal the functional ingredients and their related mechanisms to optimize and screen formulations rationally.

## Methods

### Animals and Groups

Animal experiments were permitted by the Inner Mongolia Medical University Animal Ethics Committee (YKD202101074) and complied with the guidelines for animal protection and welfare regulated by the Chinese government. C57BL/6 J mice (6–8-week-old male) were obtained from Beijing Sibeifu Biotechnology Co. Ltd. (110,324,220,103,173,451). Mice were held in a clean animal laboratory for 12 h with alternating light and dark conditions. The room temp was held between 23 and 25 °C. The mice were fed ad libitum with food and water, and following 1 week of adaptive nurturing, they were randomly grouped.

Experiment 1: The groupings are as follows.

1. A control group without any intervention (C-group), (2) a POCD model group with intracerebroventricular injection of lipopolysaccharide and nephrectomy. (M-group), (3) a POCD group with dexmedetomidine treated for positive control (D-group), (4) a POCD group with EW treated (Z-group), (5) a POCD group with EW formulation treated (J-group).

Experiment 2: The groupings are as follows.

- (6)a POCD group with TAK-242 treated (T-group), (7) a POCD group with EW and TAK-242 treated (TZ-group), and (8) a POCD group with EW formulation and TAK-242 treated (TJ-group) (see Table 1).

**Table 1:** Specific grouping and drug administration

Group	Number	Dispose	Medicine	Doses	*Delivery way*
C	12	-	Saline	Equivalent volume/kg day	Intragastric administration
M	12	2 ug LPS(i.c.v.) + Unilateral nephrectomy	-	-	**-**
D	12	2 ug LPS(i.c.v.) + Unilateral nephrectomy	Dexmedetomidine	20 µg/kg	Intraperitoneal injection
Z	12	2 ug LPS(i.c.v.) + Unilateral nephrectomy	EW formulation	0.86 g/kg day	Intragastric administration
J	12	2 ug LPS(i.c.v.) + Unilateral nephrectomy	EW basic formulation	0.86 g/kg day	Intragastric administration
T	12	2 ug LPS(i.c.v.) with 1 ug TAK242(i.v.c.) + Unilateral nephrectomy	-	-	-
TZ	12	2 ug LPS(i.c.v.) with 1 ug TAK242(i.v.c.) + Unilateral nephrectomy	EW formulation	0.86 g/kg day	Intragastric administration
TJ	12	2 ug LPS(i.c.v.) with 1ug TAK242(i.v.c.) + Unilateral nephrectomy	EW basic formulation	0.86 g/kg day	Intragastric administration

*i.c.v,* intracerebroventricular injection, *EW* Mongolian medicine Eerdun Wurile

### Animal Model of Post-operative Cognitive Dysfunction

**Scheme 1:**
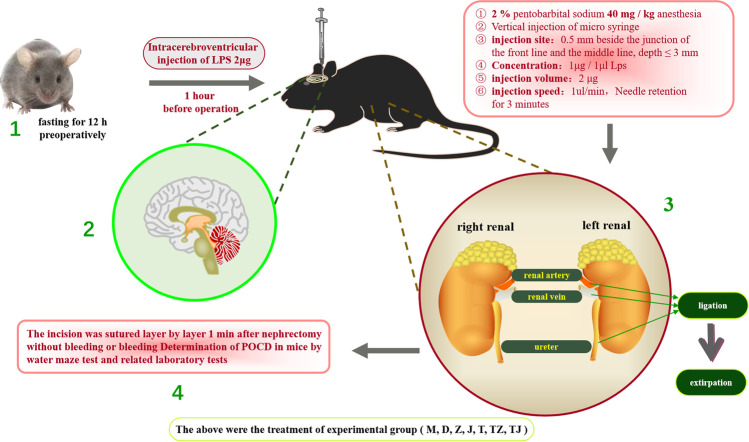
Schematic diagram of the mice POCD model

The POCD model was established after the mice fasting for 12 h on the day of surgery, and anesthesia with sodium pentobarbital (2%, 40 mg/kg) was given. When the mice are completely anesthetized, 2 μg LPS (1 μg/μL) (MedChemExpress, L2630-25MG) was administrated into the lateral ventricle with a 10-μL microsyringe. One hour after intraventricular injection of LPS, again, anesthesia with sodium pentobarbital (2%, 40 mg/kg) was given to mice and the left nephrectomy was operated. Post-operatively, the mice was analgesia with lidocaine gel. After the mice woke up, they were kept alone to recover their strength, fed soft food, and the rearing environment was the same as before surgery (see schematic diagram of the mice POCD model; Scheme 1).

### Drug Treatment

EW formulation (Fuxin Mongolian Medicine Co., Ltd., Chinese medicine approval: Z21020292) and EW basic formulation (Kulun Mongolian Medicine Factory, formula no. 111717) were each powdered and dissolved in distilled water. Six days before and on the day of operation, animals were administered intra-gastrically at 0.86 g/kg every day for 7 days; the TLR4 inhibitor TAK-242 (MedChemExpress, HY-11109) was administrated into the lateral ventricles with a 10-μL microsyringe. That is, TAK-242 (5 mg) was dissolved in the vehicle (5 mL, saline:DMSO = 9:1) and slowly administrated into the lateral ventricles at a 1-μL/min speed. The injection method is the same as intraventricular injection of LPS.

### Morris Water Maze

After 1 week of adaptive feeding, positioning and navigation training in the Morris water maze were conducted for 5 consequent days. All mice were positioned daily from quadrant 1 to quadrant 4 of the water maze and asked to search for a hidden platform. Mice searched for the platform for 60 s at a time, and if they were not on the platform within a specific time, the mice were led to the platform and observed for 10 s of memory platform positioning. The video analysis system automatically records the swimming speed and escape latency of the mice during the incubation period. For three consecutive days after surgery, a quadrant was randomly selected, the face wall of the mouse was placed in the water maze, position navigation experiments were performed, and swimming speed and escape latency were documented. We then removed the hidden platform, randomly selected a quadrant, placed the mouse in the water maze container, performed a spatial exploration experiment, and recorded the number of mice that crossed the platform and the proportion that stayed in the target quadrant within 60 s.

### Hematoxylin–Eosin Staining “”(H&E)

Mice were anesthetized with sodium pentobarbital (2%, 40 mg/kg), fix hippocampal tissue with 4% paraformaldehyde for 48 h, and paraffin wax embedding was performed. Prepare 4-μm paraffin slices, then stained with hematoxylin and eosin. Changes in mouse hippocampal tissue were detected under a microscope.

### Immunohistochemical Staining

Brain tissue was dehydrated, encapsulated, and made into serial wax sections. The sections are washed sequentially with xylene, anhydrous ethyl alcohol, gradient ethyl alcohol, and distilled water. The processed sections were then placed in a staining box with pH 8.0 EDTA antigen retrieval solution and treated to antigen retrieval process in a microwave. The serum blocking solution was added on the sections. Following removal of the blocking solution, primary antibodies, MyD88 (sc-74532), TLR4 (sc-293072), iNOS (sc-7271), NF-κB (sc-8008), and IBA-1 (sc-32725), all from Santa Cruz, were diluted as working concentrations 1:100 with the blocking solution and added to the sections at 4 °C overnight. Following incubation, tissue sections were removed and goat anti-mouse IgG conjugated with HRP secondary antibody (SeraCare, 5220–0341, 1:200) was added and room temp incubation was done for 50 min. Tissue sections were then reacted with DAB chromogenic agent at room temp for 50 min, and color development was checked by a microscope. After coloration, rinse with distilled water or tap water, hematoxylin staining was done and a microscope was used for observation. Imaging analysis was performed by ImageJ software.

### Western Blotting

MyD88, TLR4, and NF-κB expressions were assessed by Western blotting protocol. Tissue samples were incubated in RIPA lysis buffer for extraction of proteins, and the concentration of protein was assessed in each sample. After electrophoresis of the lysates by SDS-PAGE, the protein transfer was done to membranes (PVDF) previously activated with methyl alcohol. The immobilized membrane strips were incubated in the blocking solution at room temp for 1 h. After removal of the blocking solution, the diluted primary antibodies as TLR4 (Affinity, AF7017, 1:2,000), p65 (Servicebio, GB 11142, 1:1000), GAPDH (ABcam, ab8245, 1:6000), and MyD88 (ABclonal, A 16889, 1:2000) were added and left at 4 °C overnight. After primary antibodies, 5 min washing was done 3 times with TBST diluted secondary antibodies by blocking solution as goat anti-rabbit IgG conjugated with HRP (KPL, 074–1506, 1:5000) and goat anti-mouse IgG conjugated with HRP (KPL, 074–1806, 1:5000) were used for 30 min incubation at room temp. After secondary antibodies, membranes were placed on a shaker and incubated 4 times at room temp with TBST. ECL mixture solution was freshly prepared and used for exposure of the membranes at the protein side in the dark. Chemiluminescence exposure time was adjusted according to the different signal levels. The film scanning was done, and the target bands were assessed by the optical density with ImageJ software.

### ELISA

After the blood was taken from the mouse orbit, it was left for 1–2 h and centrifuged at 3000 P/m for 15 min. The serum was collected, and levels of IL-1β, TNF-α, and IL-6 were determined by ELISA kit (Wuhan Jimei Biotechnology Co. LTD). The procedures were performed according to the manufacturer’s instructions.

### RT-qPCR

MyD88, TLR4, and NF-kB expression levels were measured by reverse transcriptase (RT) quantitative PCR (RT-qPCR). Homogenized tissues were used for total RNA extraction with reference to a kit (Seville Biotechnology Co., Ltd.). RT reaction was then performed in a PCR machine (Kubo Technology, model: system). Three PCR tubes (0.2 mL) were set up for each RT reaction, and PCR amplification was performed (see Table 2).

**Table 2:** Sequences of mice-specific primer uses in RT-qPCR for TLR4, NF-κB, and MyD88

Genes	Primers
TLR4	F: 5′-TCCCTGCATAGAGGTGTGAAA-3′
	R: 5′-TCCACAGCCACCAGATTCTC-3′
NF-κB	F: 5′-AGGAGCAGGACATGGGATTTC-3′
	R: 5′-CCAAGTGCGAGGTGTCTGATA-3′
MyD88	F: 5′-TGCCAGCGAGCTAATTGAGAA-3′
	R: 5′-CTTCTGTTGGACACCTGGAGA-3′
GAPDH	F: 5′-AACTTTGGCATTGTGGAAGG-3′
	R: 5′-ACACATTGGGGGTAGGAACA-3′

### Data Analysis

Statistical assessment was executed by SPSS 26.0 statistical software. Obtained values were evaluated for normality and homogeneous variance, and normally distributed values were denoted as mean ± SD. The comparison by Student’s *t*-test was done for two groups without correspondence. Two-way analysis of variance (ANOVA) and Bonferroni post hoc test were utilized for multiple group comparisons at *P* < 0.05 as a statistical significance set.

## Results

### Experiment 1: Mongolian Medicine EW Formulation and Basic Formulation Can Improve POCD

**Fig. 1:**
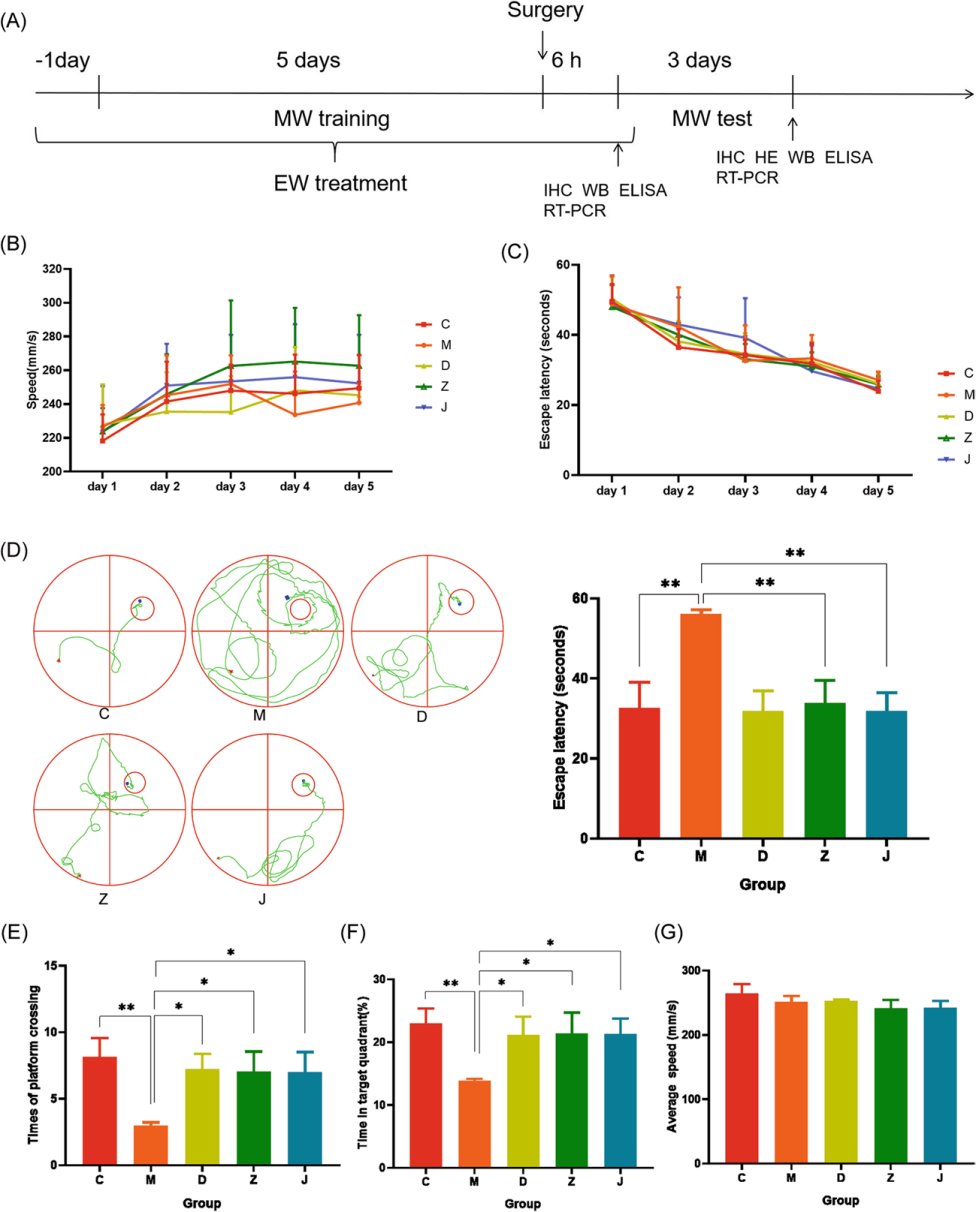
EW administration improved cognitive performance in POCD mice. **A** Schematic cartoon showing the time series of Morris water maze training, post-operative examination, and EW administration. **B** Velocity during 5 days of training during the acquisition phase. **C** Time (latency) needed for animals to reach the hidden platform during the 5-day acquisition phase training. **D** Swimming path and hidden platform training trials to assess memory and **E**, **F** spatial exploration experiments to assess learning. **G** Mean swimming speed during Morris water maze training (each data is mean ± SD, and *P < 0.05 and ** *P* < 0.01)

To determine whether the EW and EW basic formulation could ameliorate cognitive dysfunction in mice after surgery, we performed a behavioral test using the Morris water maze (Fig. 1A). Five days prior to the operation, the escape latency of each group of mice resulted in no statistically significant differences (*P* > 0.05). Compared with the first day, the mean escape latency for each group on the fifth day was shorter (*P* < 0.05) (Fig. 1C). Compared with group C, post-operative escape latency was prolonged in group M, and the times of crossing platform and the rate of stay in the target quadrant decreased (*P* < 0.05). By contrast, post-operative escape latency was shorter in groups Z, J, and D, and the times of crossing the platform and the percentage of mice staying in the target quadrant increased, compared with group M (*P* < 0.05) (Fig. 1D, E, F). No statistically significant differences were shown in swimming speed between the groups before and after the operation (*P* > 0.05) (Fig. 1B, G).

### Mongolian Medicine’s EW Formulation and Basic Formulation Reduces CNS Inflammation in POCD Mice via IBA-1, IL-6, IL-1β, TNF-α, and iNOS

#### Results of H&E Staining

**Fig. 2:**
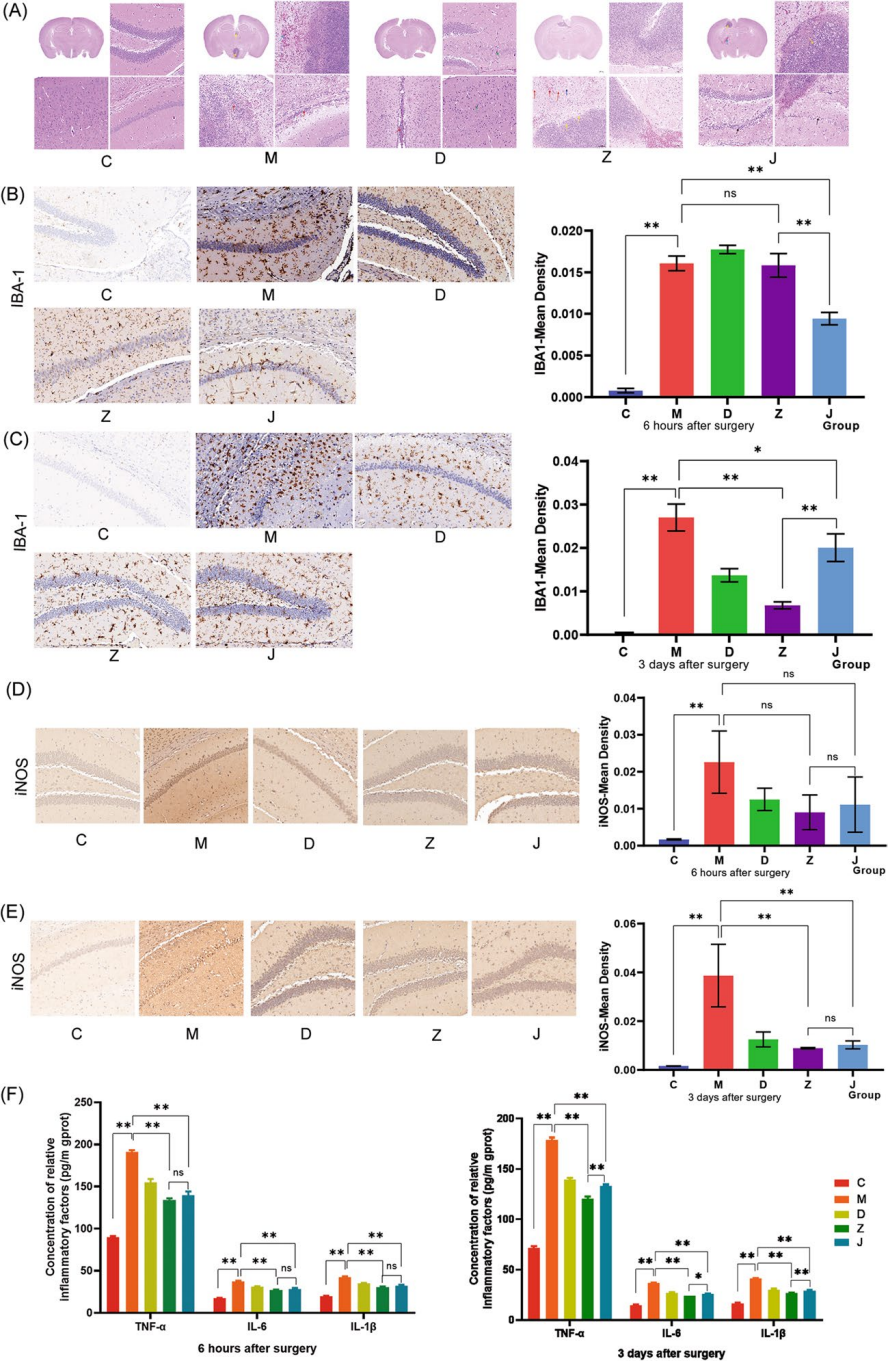
Neuroinflammation was reduced by EW treatment in the POCD mouse hippocampus. **A** H&E staining of the brain. **B**, **C** IHC staining of IBA-1-positive region of the hippocampus and statistical graph. **D**, **E** IHC staining and statistical graph of the iNOS-positive region of the hippocampus. **F** Measurement of IL-6, IL-1β, and TNF-α in serum by ELISA. Data denote mean ± SD, and *P < 0.05 and **P < 0.01

H&E staining was used to detect the effects of intracerebroventricular injection of lipopolysaccharide combined with unilateral nephrectomy on hippocampal tissue in mice (Fig. 2A).

### Immunohistochemical Results

IBA-1 was used to label microglial activation in the mouse hippocampus; compared with group C, the number of labeled microglia in group M increased at 6 h and 3 days post-operation (*P* < 0.05). On the other hand, the number of labeled hippocampal microglia reduced in group J compared with group M at 6 h and 3 days post-operation (*P* < 0.05). Compared with group M, no statistically significant differences were detected in the activation of hippocampal microglia in group Z at 6 h post-operation (*P* > 0.05), but in group Z, the number of labeled microglia in the hippocampus at 3 days post-operation decreased (*P* < 0.05) (Fig. 2B, C).

iNOS expression in the hippocampus of each group was compared at 6 h and 3 days post-operation. Compared with group C, the iNOS expression in the group M hippocampal tissue was up-regulated at 6 h and 3 days post-operation (*P* < 0.05). Compared with group M, no statistically significant differences were detected in groups D, Z, and J at 6 h post-operation (*P* > 0.05), but it decreased significantly at 3 days post-operation decreased (*P* < 0.05) (Fig. 2D, E).

#### ELISA Results

The inflammatory cytokine expression of IL-1β, TNF-α, and IL-6 in the serum of mice in each group was detected: Compared with group C, IL-1β, TNF-α, and IL-6 expressions increased at 6 h and 3 days post-operation in group M (*P* < 0.05), while compared with group M, these expressions decreased at 6 h and 3 days post-operation in groups Z and J (*P* < 0.05) (Fig. 2F), suggesting that EW formulation and EW basic formulation can inhibit CNS inflammatory responses in POCD mice via IL-1β, IBA-1, TNF-α, IL-6, and iNOS.

### Mongolian Medicine EW Formulation and Basic Formulation Can Prevent the TLR4/NF-κB Pathway Activation

#### Immunohistochemical Results

**Fig. 3:**
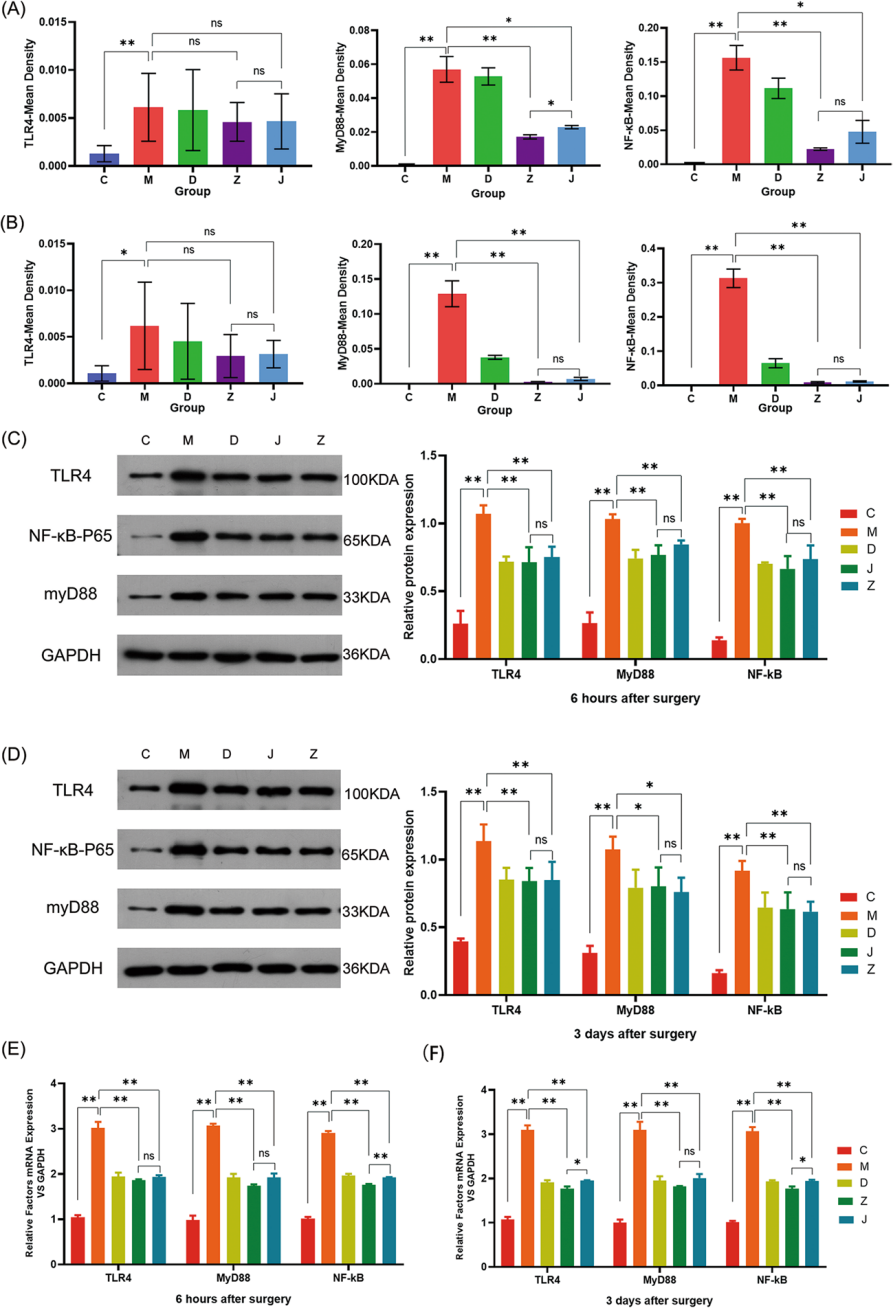
EW treatment inhibited TLR4/NF-κB activation. **A**, **B** IHC staining for TLR4/NF-κB levels in the hippocampus. **C**, **D** Western blotting of TLR4NF-κB in hippocampus. **E** RT-qPCR of TLR4NF-κB in hippocampus. Data denote mean ± SD, and *P < 0.05 and **P < 0.01

The protein expressions related to the TLR4/NF-κB in the murine hippocampus were detected by immunohistochemistry. Compared with group C, the TLR4, MyD88, and NF-κB expressions in group M were significantly up-regulated at 6 h and 3 days post-operation (*P* < 0.05) By contrast, compared with group M, the TLR4 protein expression in groups Z and J showed no statistically significant differences at 6 h and 3 days post-operation (*P* > 0.05), while NF-κB and MyD88 protein expression decreased at 6 h and 3 days post-operation (*P* < 0.05) (Fig. 3A, B).

#### Western Blotting Results

Western blotting results indicated that MyD88, NF-κB, and TLR4 expression in group M was up-regulated compared with group C at 6 h and 3 days post-operation (*P* < 0.05), consistent with immunohistochemistry results, while TLR4, MyD88, and NF-κB protein expressions in groups Z and J were reduced, compared with group M at 6 h and 3 days post-operation (*P* < 0.05) (Fig. 3C, D).

#### RT-qPCR Results

The TLR4/NF-κB activation was detected by RT-qPCR. Compared with group C, MyD88, NF-κB, and TLR4 expression in group M increased at 6 h and 3 days post-operation (*P* < 0.05). These expressions in groups Z and J decreased at 6 h and 3 days post-operation (*P* < 0.05), compared with group M (Fig. 3E, F), suggesting that the EW formulation and the EW basic formulation prevent TLR4/NF-κB activation.

### Experiment 2: The Mongolian Medicine EW Formulation and EW Basic Formulation Inhibit the TLR4/NF-κB Pathway, a Critical Mechanism for Improving POCD in Mice.

#### Results of the Morris Water Maze

**Fig. 4:**
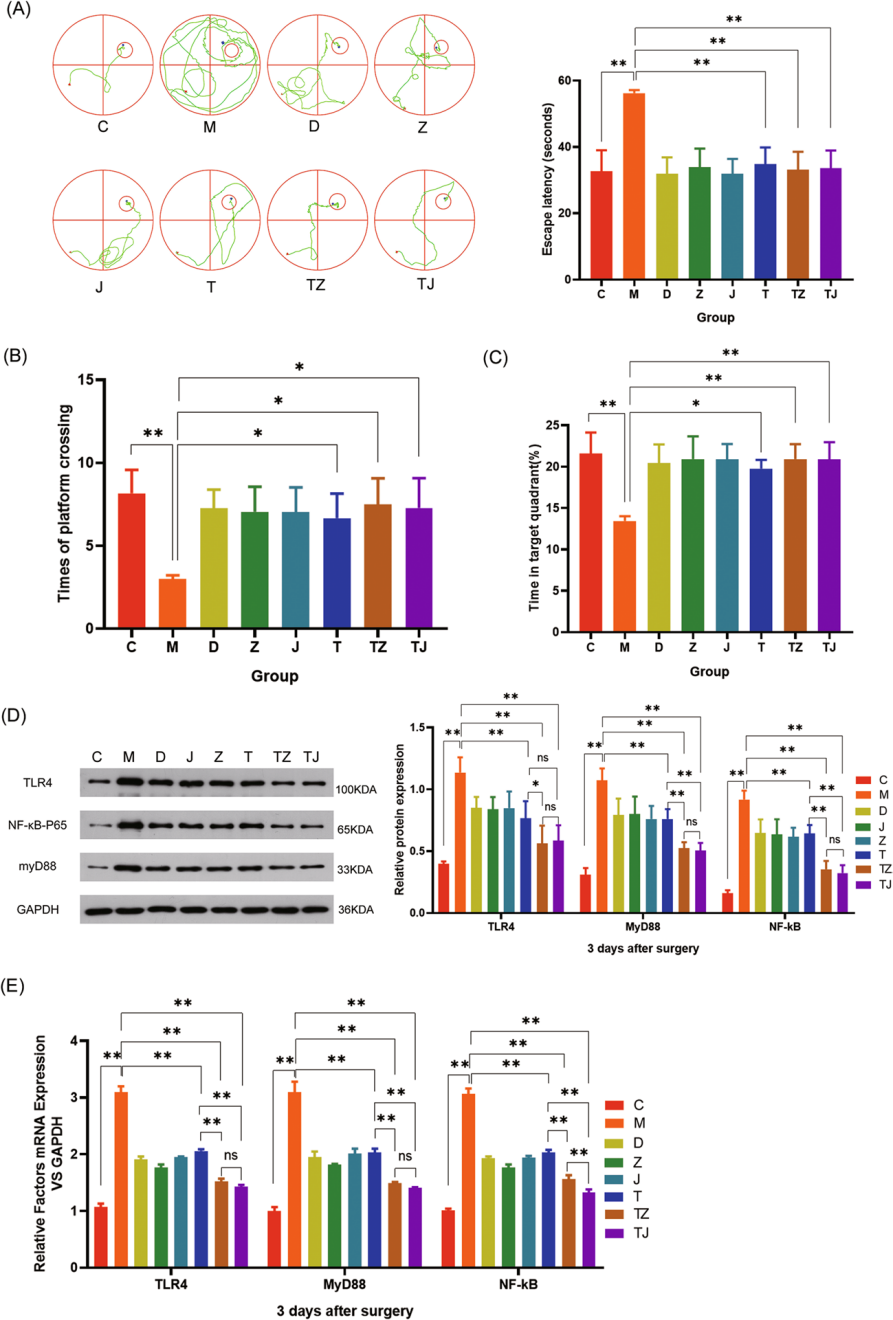
The TLR4/NF-κB is critical for EW to improve POCD in mice. **A** Western blotting of TLR4/NF-κB in the hippocampus. **B**, **C**, **D** Memory was assessed in the hidden platform training test and learning was assessed in the spatial exploration experiment. Data denote mean ± SD (*n* = 6), and *P < 0.05 and **P < 0.01

The Morris water maze was utilized to detect cognitive function in groups T, TZ, and TJ. The data indicated that, compared with the M group, the T, TZ, and TJ groups had reduced escape latencies, a higher number of crossing platforms, and higher percentages of staying in the target quadrant at each post-operative time point (*P* < 0.05), but there were not statistically significant in behavior among the T, TZ, and TJ groups (*P* > 0.05) (Fig. 4A, B, C).

#### Western Blotting Results

TAK-242, an TLR4/NF-κB inhibitor, was applied to POCD mice. The MyD88, NF-κB, and TLR4 expressions in the hippocampus of T, TZ, and TJ groups were assessed by Western blotting on the third post-operative day. Compared with the M group, the MyD88, NF-κB, and TLR4 expressions in the T, TZ, and TJ groups were reduced (*P* < 0.05). Compared with the T group, the TLR 4, MyD88, and NF-κB expressions decreased in TZ group (*P* < 0.05), while MyD88 and NF-κB expressions decreased in the TJ group (Fig. 4D).

#### RT-qPCR Results

The RNA expressions of MyD88, NF-κB, and TLR4 in the hippocampus of T, TZ, and TJ groups were detected by RT-qPCR on the third post-operative day. The data showed that MyD88, NF-κB, and TLR4 expressions in the T, TZ, and TJ groups were down-regulated at the expression levels compared with the M group (*P* < 0.05), and TLR4, MyD88, and NF-κB expressions in the TZ and TJ groups decreased compared with the T group (*P* < 0.05) (Fig. 4E), suggesting that suppressing TLR4/NF-κB activation is an essential mechanism for EW formulations and basic formulations in improving POCD in mice.

## Discussion

Although the precise pathogenesis of POCD is still unclear, several investigations have verified that inflammatory cytokine-related mechanisms are involved in critical roles in the development and regression of POCD [4, 5, 21]. As immune effector cells are intrinsic to the central nervous system, microglia are known to mediate CNS inflammation [22]. IBA-1 was used to label microglia in the murine hippocampus, and compared with the M group, microglial activation in the hippocampal tissues of the Z and J groups was substantially suppressed on post-operative day 3 (*P* < 0.05). Inflammatory factors may stimulate iNOS expression and release large amounts of NO. High concentrations of NO are cytotoxic, directly damaging DNA and mitochondrial function and impairing cells [23, 24]. We detected iNOS expression in the hippocampus of groups C, D, M, Z, and J. Compared with group C, iNOS in hippocampus of group M was significantly increased (*P* < 0.05), while EW formulation and basic formulation significantly inhibited the expression of iNOS on post-operative day 3 (*P* < 0.05). Lipopolysaccharide is a primary element of Gram-negative micro bacteria’s cell wall and binds specifically to TLR4, which can stimulate the NF-κB and produce inflammation in the central nervous system. At the same time, lipopolysaccharide can disrupt the blood–brain barrier and cause memory impairment [25]. Several studies have confirmed that lipopolysaccharide causes a peak in inflammation 6 h after it enters the body [26], while the peak of POCD in mice after surgical stress is usually observed on the third day after surgery [27]. In this experiment, we found the inflammatory factor expressions in each group’s serum. Compared with 6 h post-operation, no statistically significant differences were shown in IL-1β and IL-6 expressions in group M on a post-operative day 3 (*P* > 0.05) but IL-1β, IL-6, and TNF-α expressions in groups Z and J decreased on a post-operative day 3 (*P* < 0.05), suggesting that the EW formulation and basic formulation may improve the POCD development in mice by suppressing inflammatory responses in vivo.

EW has anti-inflammatory, antioxidant, and free radical scavenging properties [28, 29]. Ten drugs from EW formulation (Margarita, Glycyrrhizae radix et rhizoma, Inulae radix, Aucklandiae radix, Aquilariae lignum resinatum, Bovis calculus artifactus, Piperis longi fructus, Euphorbiae humifusa herba, Powdered buffalo horn extract, and Moschus) were selected to form the EW basic formulation. Analysis of the Morris water maze data indicated that compared with the M group, the murine behavioral results in the Z and J groups were substantially improved (*P* < 0.05). By contrast, no statistically significant differences were detected between the J and Z groups (*P* > 0.05). Compared with group Z, no statistically significant differences were shown in IL-1β, TNF-α, and IL-6 expressions at 6 h post-operation in group J (*P* > 0.05), but microglial activation was decreased (*P* < 0.05). On post-operative day 3, IL-1β, TNF-α, IL-6 expressions, and microglial activation up-regulated in the J group (*P* < 0.05), suggesting that the basic formulation of EW can improve POCD in mice, but the specific mechanism and effective ingredients of EW formulation in improving POCD still need to be further investigated.

Currently, no specific drugs for treating POCD are identified. Dexmedetomidine is currently recognized as an ameliorating agent for POCD [30]. In this experiment, no statistically significant differences were detected in behavioral test results between groups D, Z, and J (*P* > 0.05). Compared with group D, group Z had decreased IL-1β, TNF-α, and IL-6 expression levels at 6 h and 3 days post-operation (*P* < 0.05). Compared with group D, IL-1 β, IL-6, and TNF-α expressions decreased in group J at 6 h post-operation (*P* < 0.05) and TNF-α at 3 days post-operation (*P* < 0.05), but IL-6 and IL-1 β expressions at 3 days post-operation were not statistically significant (*P* > 0.05). The results of this experimental study show that the efficacy of EW and EW basic formulation in the treatment of POCD is comparable to that of dexmedetomidine in the treatment of POCD. If the experimental results are translated into clinical practice, whether the two therapeutic effects can also be consistent, or whether this is related to the time and duration of the administration still needs to be further investigated.

Western blotting and RT-qPCR data indicated that MyD88, NF-κB, and TLR4 expression levels in the TZ and TJ groups on post-operative day 3 were reduced compared with the Z and J groups (*P* < 0.05), but behavioral tests showed no statistically significant differences (*P* > 0.05), suggesting that the combination of EW and the TLR4 inhibitor TAK-242 did not enhance the POCD-improving effect of the EW formulation and the basic formulation, and that it may be associated with improvement in cognitive impairment due to multi-targeting with EW.

There are limitations to this experiment: The ten drugs that comprise the basic EW formulation are effective in treating nervous system disorders. For the rigor of the experiment, the drug composition of the formulations must be analyzed to accurately determine the active ingredients related to the nervous system. This is an area to be restored in the later stages of this study.

EW, one of the treasures in the history of Mongolian medicine, can improve not only POCD in mice but also neuronal behavioral performance in a rat injury model with middle artery occlusion-reperfusion by inhibiting neuronal apoptosis, promoting neurotransmitter transmission and repairing neuronal damage. Therefore, in addition to its simple anti-inflammatory effects, other mechanisms of EW in improving post-operative cognitive dysfunction may be worth further investigation.

## Conclusions

The Mongolian medicine EW formulation and EW basic formulation can improve postoperative cognitive dysfunction in mice, and TLR4/NF-κB pathway seems to be one of the important mechanisms in EW’s improvement of POCD.

## Data Availability

Original contributions to the study are included in the article/supplementary material; for further inquiries, the corresponding authors can be contacted.
